# FMISO-PET-derived brain oxygen tension maps: application to glioblastoma and less aggressive gliomas

**DOI:** 10.1038/s41598-017-08646-y

**Published:** 2017-08-31

**Authors:** Ararat Chakhoyan, Jean-Sebastien Guillamo, Solène Collet, François Kauffmann, Nicolas Delcroix, Emmanuèle Lechapt-Zalcman, Jean-Marc Constans, Edwige Petit, Eric T. MacKenzie, Louisa Barré, Myriam Bernaudin, Omar Touzani, Samuel Valable

**Affiliations:** 1 Normandie Univ, UNICAEN, CEA, CNRS, ISTCT/CERVOxy group, 14000 Caen, France; 2 CHU de Caen, Service de Neurologie, 14000 Caen, France; 3 CNRS, UMR6139 LMNO, 14000 Caen, France; 4 CNRS, UMS3408, 14000 Caen, France; 5 CHU de Caen, Service d’Anatomie-Pathologique, 14000 Caen, France; 6 CHU de Caen, Service de Radiologie, 14000 Caen, France; 7 Normandie Univ, UNICAEN, CEA, CNRS, ISTCT/LDM-TEP group, 14000 Caen, France

## Abstract

Quantitative imaging modalities for the analysis of hypoxia in brain tumors are lacking. The objective of this study was to generate absolute maps of tissue p_t_O_2_ from [^18^F]-FMISO images in glioblastoma and less aggressive glioma patients in order to quantitatively assess tumor hypoxia. An ancillary objective was to compare estimated p_t_O_2_ values to other biomarkers: perfusion weighted imaging (PWI) and tumor metabolism obtained from ^1^H-MR mono-voxel spectroscopy (MRS). Ten patients with glioblastoma (GBM) and three patients with less aggressive glioma (nGBM) were enrolled. All patients had [^18^F]-FMISO and multiparametric MRI (anatomic, PWI, MRS) scans. A non-linear regression was performed to generate p_t_O_2_ maps based on normal appearing gray (NAGM) and white matter (NAWM) for each patient. As expected, a marked [^18^F]-FMISO uptake was observed in GBM patients. The p_t_O_2_ based on patient specific calculations was notably low in this group (4.8 ± 1.9 mmHg, p < 0.001) compared to all other groups (nGBM, NAGM and NAWM). The rCBV was increased in GBM (1.4 ± 0.2 when compared to nGBM tumors 0.8 ± 0.4). Lactate (and lipid) concentration increased in GBM (27.8 ± 13.8%) relative to nGBM (p < 0.01). Linear, nonlinear and ROC curve analyses between p_t_O_2_ maps, PWI-derived rCBV maps and MRS-derived lipid and lactate concentration strengthens the robustness of our approaches.

## Introduction

Hypoxia is a critical component of the glioblastoma (GBM) microenvironment and has been associated with both poor prognosis and resistance to various therapies^1^. There is a marked correlation between hypoxia, vascular dysfunction and tumor aggressiveness^2^. It is also known, since the 1950’s, that the low oxygenation observed in the tumor plays a major role in the resistance to X-ray radiation therapy^3^. The conventional treatment of GBM is surgical resection when possible, followed by radiotherapy along with concomitant chemotherapy. Despite the rigorous treatment protocol, the median survival fails to exceed 15 months^4^. Multi-parametric MRI or CT imaging are routinely used for tumor diagnosis, for the planning of radiation treatment but also for tumor follow-up after treatment. However, molecular imaging with positron emission tomography (PET) tracers sensitive to various biological parameters have now enlarged the range of functional information available for more targeted treatments and treatment efficacy determination^5^. For instance, biological target volume (BTV) used in radiotherapy could provide additional relevant data for dose painting in biologically active sub-regions or to boost radiotherapy^6^. Several approaches have been published over the last decade to estimate tumor hypoxia, or tumor oxygenation, including Eppendorf probes, electron paramagnetic resonance (EPR), ^19^F-MRI, MRI quantitative BOLD and another recently developed MRI technique based on lipid relaxation enhancement termed MOBILE [for review see Corroyer-Dulmont *et al*., 2015]^7^. Until now, PET imaging has been demonstrated to be the most rigorous (and “gold standard”) approach to map tumor hypoxia. Yet, PET is a quantitative imaging technique and remains the most sensitive and specific approach. One of the most common hypoxia-specific tracers in PET imaging is 3-[18 F]-fluoro-1-(2-nitro-1-imidazolyl)-2-propanol ([^18^F]-FMISO)^8^. However, it is well known that the relationship between oxygen tension and [^18^F]-FMISO uptake is non-linear^9^ and estimation of p_t_O_2_ (oxygen pressure in the tissue) remains mandatory for advanced use of FMISO maps since ptO_2_ maps are of greater biological pertinence. For instance, the accurate calculation of dose modulation for radiotherapy based on the Oxygen Enhancement Ratio effect requires ptO_2_ mapping^10^. Two main models have been proposed in the literature to convert [^18^F]-FMISO images into p_t_O_2_ maps^11, 12^. The former is a complex approach that uses many parameters to relate FMISO uptake to oxygen partial pressure but that was validated at the preclinical level. The latter is a simpler approach but needs to be validated in various situations.

To the best of our knowledge, adapted and validated mathematical models have never been employed in patients suffering from glioma. Here, we have initially employed in GBM patients the mathematical model previously used for head and neck cancers^12^. Nonetheless, this model turned out misfit. Therefore, the aim of the present study was to propose a new method of mathematical modeling to build-up p_t_O_2_ maps from [^18^F]-FMISO images for individual patient. Ten patients with GBM (GBM group) and three others patients with less aggressive glioma (nGBM group) were included and in which the proposed model was validated through the use of indirect estimates of tumor oxygenation from multimodal imaging; namely MRS and rCBV maps which were used to reflect anaerobic tumor metabolism and vascular status, respectively.

## Results

### Description of patients and images

**Figure 1:**
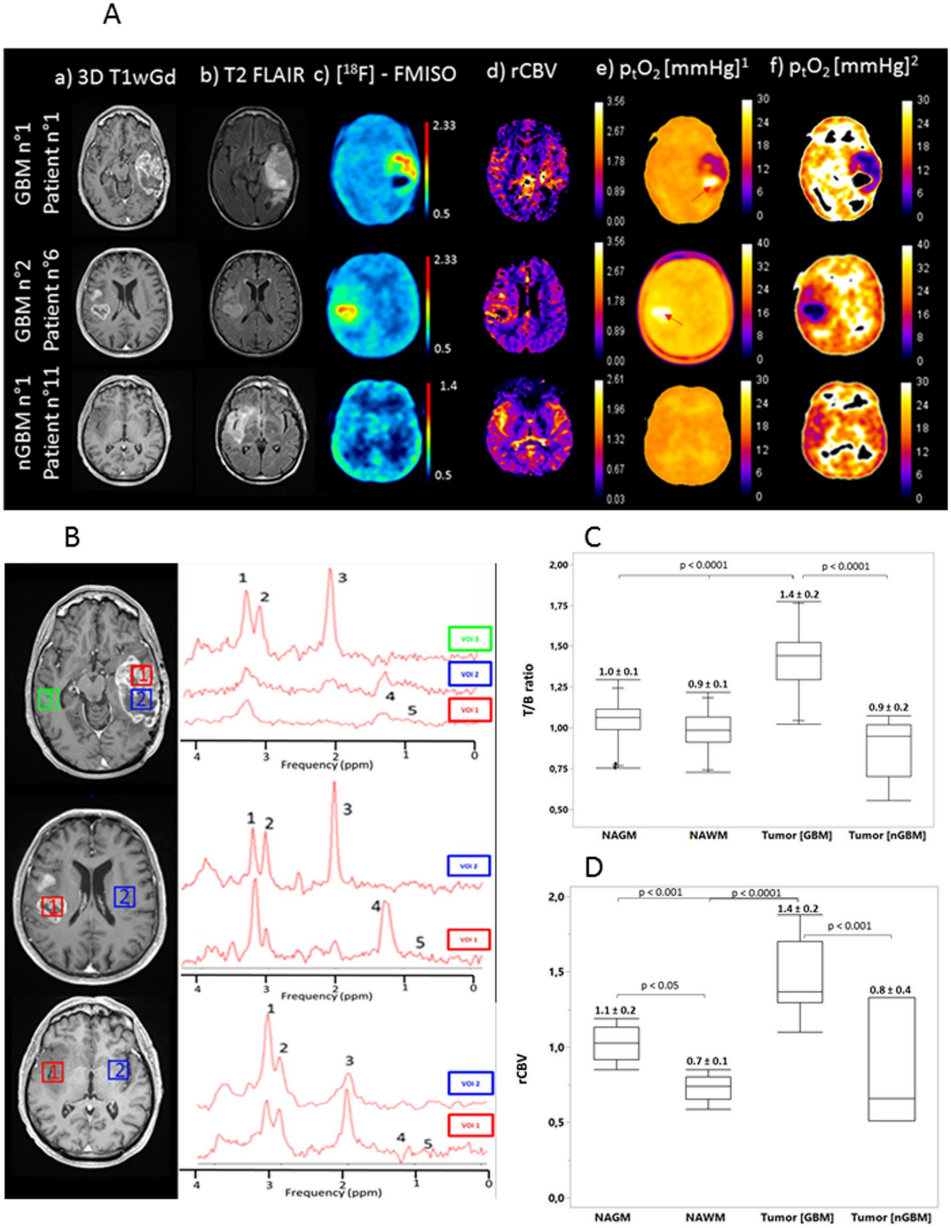
(**A)** Representative axial 3D T1wGd (a), fluid attenuation inversion recovery (FLAIR) (b), [^18^F]-FMISO (c), rCBV maps (d), representative absolute p_t_O_2_ maps (e) on the basis of the values of (a,b and c), proposed by Toma-Dasu and coll. and adjusted p_t_O_2_ (f) maps with empirical approach (non-linear regression) of two glioblastoma (GBM) and one less aggressive glioma (nGBM) patients. (**B**) ^1^H-MR mono-voxel spectroscopy was performed (Te = 144 ms) in tumor and/or necrotic tissue as well as non-tumor tissue for the same patients. Spectral peak locations and VOIS: (1) choline, (2) creatine, (3) NAA (N-acetyl aspartate, (4) lactate and (5) lipids. (**C**) Box plot of T/B ratio and rCBV (**D**) quantification in NAGM, NAWM and GBM and nGBM tumors areas.

GBM patients were characterized by the extravascular leakage of Gd-DOTA, hyperintensities on FLAIR images, a well-defined [^18^F]-FMISO uptake, an elevated rCBV and a decrease in the NAA signal concomitant to an increase in the lactate and lipid signal (Fig. 1A, two uppers panels and Fig. 1B). In contrast, nGBM were characterized by no extravasation of Gd-DOTA, hyperintensities on FLAIR, basal [^18^F]-FMISO uptake corresponding to the washout of the radiopharmaceutical in oxic conditions, nearly normal rCBV and a MRS profile compatible with that of less aggressive tumors (Fig. 1A, lower panel and Fig. 1B). The mean tumor volume based on contrast enhancement is 36.4 ± 34.7 cm^3^ in GBM group. Additionally, the edema region quantified in T2w-Flair contrast is 113.1 ± 70.0 cm^3^ and 25.8 ± 18.4 cm^3^ for both GBM and nGBM patients. Quantitatively, the ratio of [^18^F]-FMISO was 1.4 ± 0.2 for GBM and 0.9 ± 0.1 for nGBM (p < 0.001). The rCBV was 1.4 ± 0.2 for GBM and 0.8 ± 0.4 for nGBM (p < 0.001) (Fig. 1C,D).

### Estimation of p_t_O_2_

We firstly used the unknown parameters initially described^12^ to calculate the p_t_O_2_ value from the [^18^F]-FMISO uptake. With this empirical approach, the mean p_t_O_2_ in NAGM and NAWM were 19.7 ± 0.5 and 20.9 ± 0.4 mmHg.

In the contrast-enhanced tumor of GBM group, the mean p_t_O_2_ was 15.2 ± 1.1 mmHg; in the nGBM patient, the volume of edema was delineated and the mean p_t_O_2_ was found to be 19.4 ± 0.4 mmHg. Necrotic foci of the tumors in GBM patients also appeared abnormally elevated (arrows Fig. 1A(e)). Given that the retention of [^18^F]-FMISO in a hypoxic cell occurs only when the partial pressure of oxygen is less than 10 mmHg^13^ and that no [^18^F]-FMISO metabolism should have occurred in the necrotic region of the tumor as well as in the health tissue, we hypothesized that the initial parameters reported for the operational equation would require further adaptation for normal and pathological brain tissues.

### Adaptation of the model for intracerebral tissues

We consequently determined the equation by fixing the p_t_O_2_ in two regions (NAGM and NAWM) which allowed a hyperbolic function to convert the [^18^F]-FMISO uptake to absolute values of p_t_O_2_. For each patient, **a**, **b** and **c** were estimated. The mean values were 1.5, 0.7 and 9.9 respectively.

**Figure 2:**
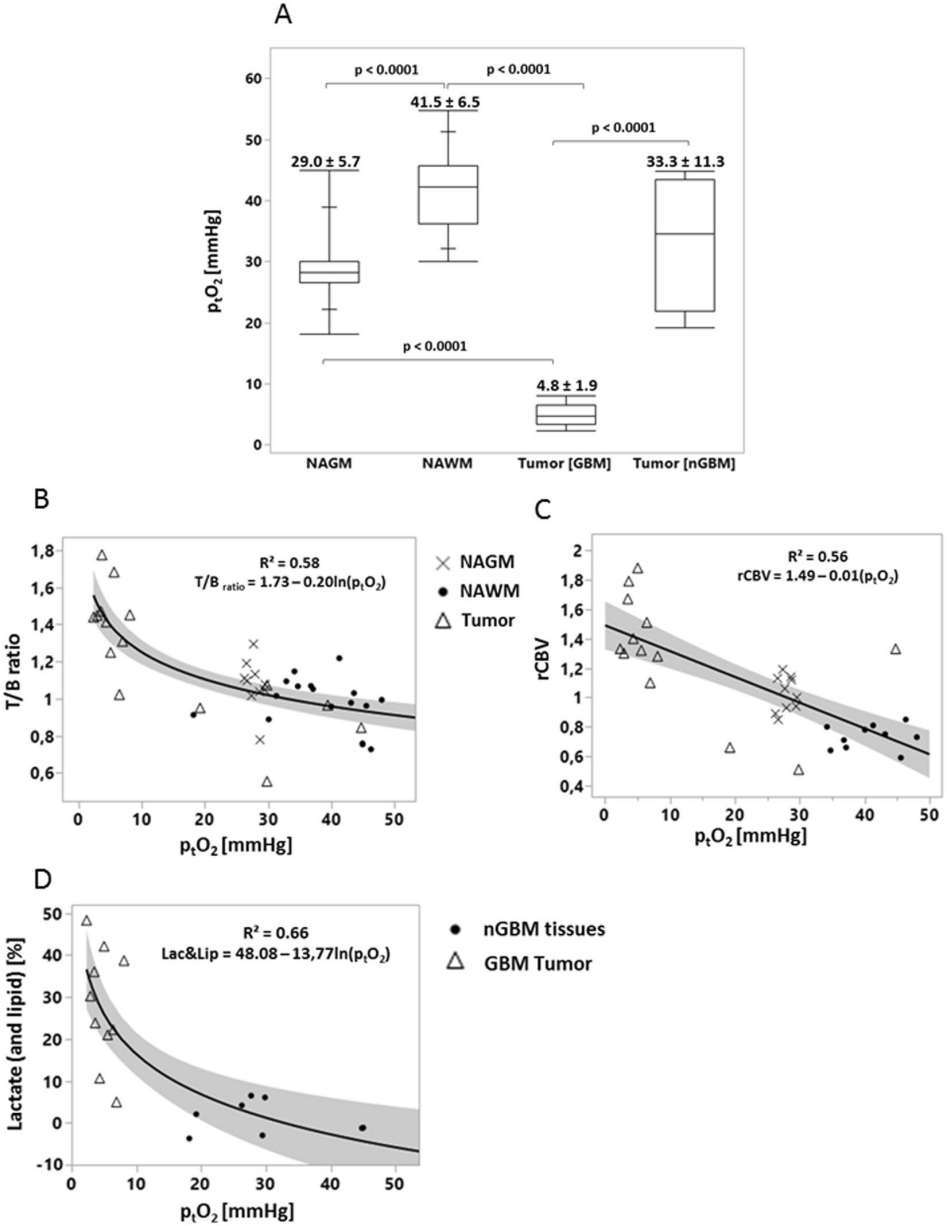
Box plot of adjusted p_t_O_2_ values (medians and the four quartiles as well as individual results) for each region of interest (NAGM, NAWM, GBM tumor and nGBM tumor) and the corresponding absolute values as mean ± sd (Fig. 2A). Correlation regressions between [^18^F]-FMISO uptake (T/B ratio), p_t_O_2_, rCBV and the lipids and lactate concentration. (**B**) Non-linear fit between [^18^F]-FMISO uptake and adjusted p_t_O_2_ values. Each point represent a volume of interest (x NAGM | • NAWM | Δ Tumor). (**C**) Linear fit between rCBV (calculated from PWI-MRI imaging) and p_t_O_2_ (estimated form [^18^F]-FMISO uptake). (**D**) Non-linear fit between Lactate and lipid [%] (calculated from MRS) and p_t_O_2_.

After fitting the data and generating p_t_O_2_ maps (Fig. 1A(f)), the mean p_t_O_2_ was 29.0 ± 5.7 and 41.5 ± 6.5 mmHg in NAGM and NAWM, respectively (p < 0.001). The mean estimated p_t_O_2_ in the tumors was 4.8 ± 1.9 mmHg in the GBM group and 33.3 ± 11.3 mmHg for the nGBM group (p < 0.001) (Fig. 2A). No significant differences were found between the oxygen status in NAGM and nGBM tissues.

### Comparison of p_t_O_2_ maps with rCBV maps and ^1^H-MRS metabolites

Linear and logarithmic regressions were applied between [^18^F]-FMISO, p_t_O_2_, rCBV, lactate (and lipid) concentration (Fig. 2B–D). A significant non-linear relationship was found between the [^18^F]-FMISO uptake (T/B ratio) and affined p_t_O_2_ across tumoral, NAGM and NAWM areas (R² = 0.58) (Fig. 2B). More interestingly, in the same areas, the correlation was also demonstrated between rCBV and p_t_O_2_ (R² = 0.56) (Fig. 2C). On the other hand, the correlation between p_t_O_2_ and lactate/lipid concentrations was also significantly evidenced (R² = 0.66) (Fig. 2D).

### ROC analyses for tumor and tissue type detection

**Figure 3:**
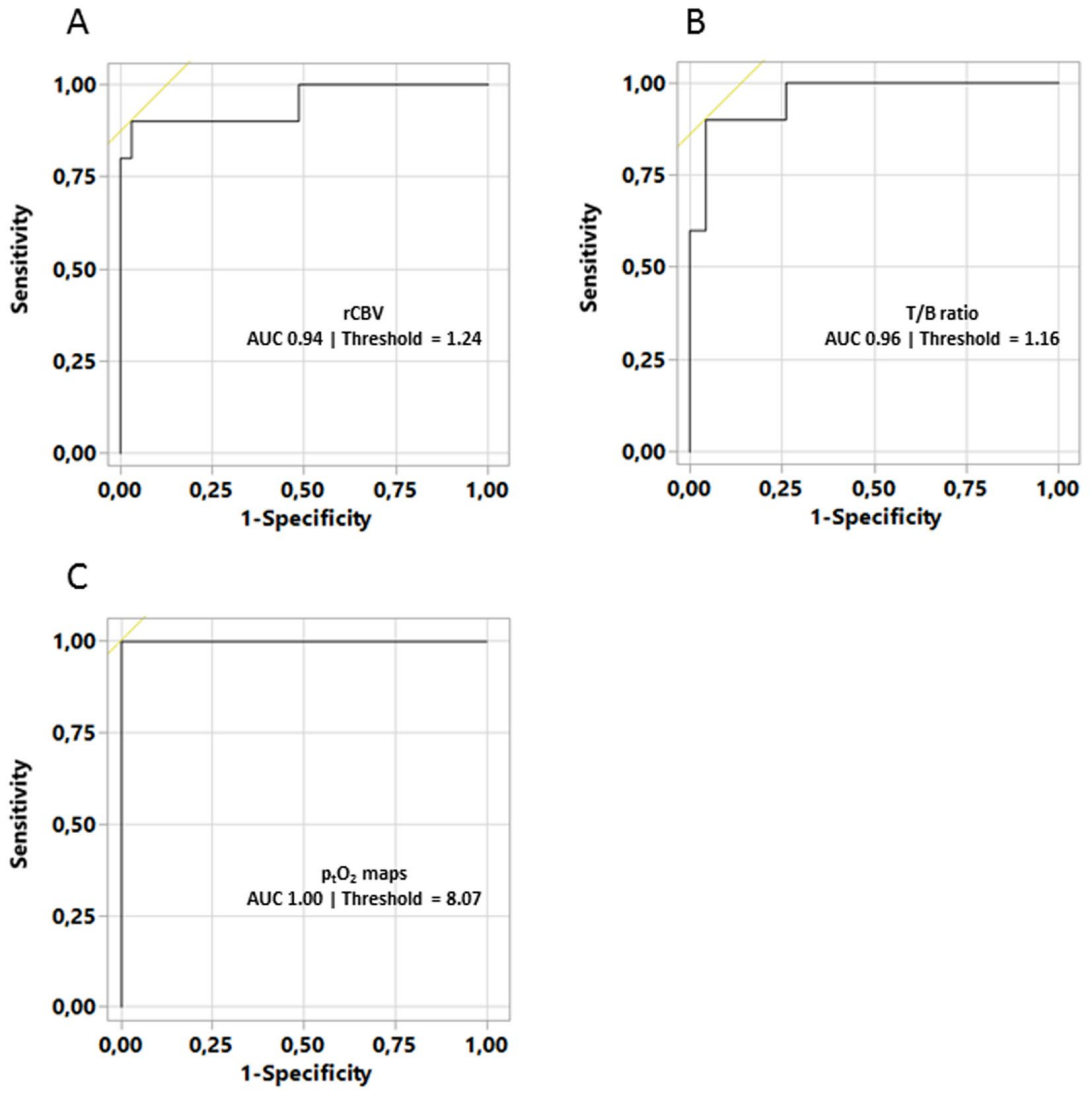
ROC curve analyses for discrimination of GBM and nGBM) using rCBV (**A**), T/B ratio maps (**B**) and affined p_t_O_2_ maps (**C**). The AUC and ROC threshold (for best sensitivity and specificity) values are reported in each ROC histogram.

The ROC curves were performed to assess the ability of the various estimates to discriminate between GBM and nGBM patients (Fig. 3). The AUC value was 0.94 for CBV (threshold = 1.24); 0.96 for [^18^F]-FMISO (threshold = 1.16). Interestingly; the AUC was 1.00 for p_t_O_2_ (threshold = 8.07 mmHg).

## Discussion

In GBM, hypoxia has been identified as an independent factor of poor prognosis^1^. We present here an approach to estimate p_t_O_2_ maps calibrated for individual patients and based on [^18^F]-FMISO PET that markedly discriminates severely hypoxic GBM from nGBM; p_t_O_2_ maps correlate well with other indices of oxygen status and may suggest avenues to explore personalized radiotherapy.

In the present study, we demonstrate that the model initially proposed by Toma-Dasu and his group^12^ for head and neck cancer to calculate intracerebral p_t_O_2_ requires further adaptation in the specific instance of brain tumors. After an *ad hoc* calculation of the parameters for individual patients based on the values of [^18^F]-FMISO retention in their corresponding NAGM and NAWM, we calculated p_t_O_2_ in NAGM as 29.0 ± 5.7 mmHg and that of NAWM as 41.5 ± 6.5 mmHg. Measurements in the tumor of GBM subjects revealed a mean p_t_O_2_ of 4.8 ± 1.9 mmHg that is in good agreement with the measurements obtained with oxygen sensitive probes^14^. However, one should mention that the assessment of tissue oxygen with probes remains problematic due to the high spatial heterogeneity observed in GBM patients. Additionally, in the nGBM group, the calculated p_t_O_2_ was 33.3 ± 11.3 mmHg in the tumoral tissue. This value is closed to that of NAGM and NAWM values and also concords with previous probe-based results^15^. Our findings clearly demonstrate the existence of severe hypoxia in GBM tumors, which may, in turn, explain the limited response to radiation therapy.

In terms of rCBV status, a marked and significant difference was observed between nGBM and GBM, which is in agreement with the literature^16^. The reproducibility of rCBV maps is reliable in non-pathological brain regions (NAGM and NAWM) in the two groups of patients (rCBV_GM_ = 1.1 ± 0.2 and rCBV_WM_ = 0.7 ± 0.1 respectively). Additionally, we failed to detect outright perfusion abnormalities in the nGBM group. In contrast, rCBV was increased in the GBM group (p < 0.05). The correlation between rCBV and p_t_O_2_ revealed an inverse linear relationship. These results confirm those of Cher and colleagues^17^ and those of Gerstner and colleagues^18^ based on histological and MRI markers of the vasculature and hypoxia assessed by [^18^F]-FMISO PET and reinforce the idea that the vessels observed in GBM are poorly functional; the result is a drastic fall in p_t_O_2_ values. We also applied the ROC curve analysis on GBM and nGBM patients discrimination, and observed that the sensitivity and specificity were improved when p_t_O_2_ was used instead of [^18^F]-FMISO and rCBV maps.

A correlation was also found between p_t_O_2_ values and the lactate and lipid concentrations, which may reflect an intense anaerobic metabolism resulting with lactate production in highly hypoxic tissues.

In the present study, we have developed a mathematical algorithm to enable the calculation of absolute p_t_O_2_ in tumoral tissues at the individual level. The values so obtained are in accordance with other, albeit indirect, estimations of oxygenation status, namely blood volume and indices of anaerobic metabolism. As radiosensitivity parameters that take into account the OER, and the non-linearity between p_t_O_2_ and [^18^F]-FMISO, we believe that the present p_t_O_2_ maps should be used rather than [^18^F]-FMISO to evaluate the impact of dose escalation on tumor control probability (TCP) models.

The principal features of our investigation are undernoted. i) The determination of the three factors necessary to calculate p_t_O_2_ from [^18^F]-FMISO maps and, these, in individual ROI and in individual subjects. ii) Our innovative approaches allow in terms of tissue oxygenation, a marked discrimination between the values in GBM (severely hypoxic) and less aggressive glioma as well as apparently normal brain tissue, be it white or gray matter. The overall findings would encourage prospective study.

However, our proof of concept has some limitations. First, the calculation of p_t_O_2_ is based on a mathematical model. Although other models have been proposed in the literature^9, 11^, the relationship between [^18^F]-FMISO uptake and p_t_O_2_ remains hyperbolic. The advantage of our approach is that it requires two regions of interest for calibration of the model.

Another limitation of this study is that [^18^F]-FMISO is trapped into hypoxic cells when p_t_O_2_ is less than 10 mmHg^19^ rendering the calculation of p_t_O_2_ less adapted for low grade gliomas.

Finally, in our small cohort, [^18^F]-FMISO uptake was only observed in GBM but not in less malignant gliomas (grade II and III). These findings concord with numerous published studies in which, GBM display invariably an increased uptake of [^18^F]-FMISO^20, 21^. Nonetheless, it is less evident that less aggressive glioma develop hypoxia. Cher and colleagues^22^ reported that all the GBM showed a high uptake of [^18^F]-FMISO, however they reported that only one patient of grade III shows a positive uptake of [^18^F]-FMISO. In another study^23^, the authors reported a positive uptake in grade III but the uptake observed in grade IV was significantly higher. Our results concord with these observations since a very severe hypoxia was obtained for GBM and not for less aggressive grades, however, our proposed model needs to be tested in a larger cohort of GBM and less aggressive gliomas. Additionally, this hypoxic marker and its conversion could be a powerful tool for stratification in radiation treatment.

In the present study, the main goal was to analyze the feasibility of adapting a model, formerly developed for head and neck to brain tumors, as a proof of concept. Future investigations are needed to test this hypothesis on the tumor control improvement with radiotherapy ought to benefit from the wealth of information based on functional imaging. Molecular imaging with positron emission tomography (PET) tracers is sensitive to various biological parameters have now enlarged the range of functional information available for more targeted treatments planning by defining a so called “ biological target volume “ (BTV) treatment efficacy determination. The BTV could provide additional relevant data for dose painting in biologically active sub-regions or to boost radiotherapy.

## Methods

### Patients

This study was part of a prospective monocentric clinical trial called HYPONCO, funded by INCa (Institut National du Cancer). The study was approved by both the ethics committee (CPP Nord-Ouest III) and AFSSAPS (Agence française de sécurité sanitaire des produits de santé) and registered under EUDRACT number 2009-015543-16. More information’s are available bellow: (NTC01200134 - https://www.clinicaltrials.gov/ct2/show/NCT01200134). All procedures performed in the study were in accordance with ethical standards of both research committees. All patients are included between September 10, 2010 and November 16, 2012 after having been fully informed of the study and provided signed, informed consent.

Ten patients with confirmed GBM (based on the classification of the World Health Organization (WHO)) and three patients with less aggressive glioma were investigated. The characteristics of the patients are summarized (Table 1). After the discovery of a glioma (based on anatomical MRI), PET imaging was employed to detect regions of hypoxia. The day following PET imaging, an MRI study (anatomic, vascular and metabolic) was performed. PET images and anatomical 3D T1w-Gd images were used to guide the placement of the volumes of interest (VOI) for MRS.

**Table 1:** Patients and tumor characteristics.

No	Sex	Age	KP	Tumor Location	CE	T/B	WHO Grade	Histology
1	M	28	100	Parietal, Temporal, Insular, L	Yes	Yes	IV	Glioblastoma
2	M	58	90	Temporal, R	Yes	Yes	IV	Glioblastoma
3	M	64	80	Frontal, L	Yes	Yes	IV	Glioblastoma
4	M	52	80	Frontal, R	Yes	Yes	IV	Glioblastoma
5	M	62	80	Occipital, CC, L	Yes	Yes	IV	Glioblastoma
6	M	67	90	Temporal, Insular, R	Yes	Yes	IV	Glioblastoma
7	M	56	70	Occipital, CC, Temporal, L	Yes	Yes	IV	Glioblastoma
8	M	63	90	Frontal, L	Yes	Yes	IV	Glioblastoma
9	F	71	70	Parietal, Occipital, R	Yes	Yes	IV	Glioblastoma
10	M	46	80	Frontal, CC, R, L	Yes	Yes	IV	Glioblastoma
11	M	51	100	Temporal, Insular, R	No	No	III	Oligoastrocytoma
12	M	58	90	Hemisphere, R, Brain-stem	Yes	No	III	Gliomatosis
13	M	54	100	Frontal, R	No	No	II	Oligodendroglioma

M: Male, F: Female, KP: Karnofsky’s performance scale, R: Right, L: Left, CC: Corpus callosum. CE: Contrast enhancement after Gadolinium, T/B: Tissue to blood ratio ≥1.2, WHO: World Health Organization.

### Positron emission tomography/CT Scan

[^18^F]-FMISO was synthesized by the LDM-TEP group (UMR6301-ISTCT, Cyceron) based on methods previously described^24^. Acquisitions performed on a General Electric Discovery VCT 64 PET/CT camera, lasted 20 min and were carried out 120 minutes after the intravenous injection of ≈5 MBq/kg of [^18^F]-FMISO. The attenuation-corrected images were reconstructed with an OSEM 2D algorithm (9 subsets and 2 iterations) and filtered in 3D with a Butterworth filter. Two blood samples were taken and the plasma averaged at the time of imaging for the calculation of T/B maps according to the following formula:

1 $${\rm{T}}{\rm{/}}{\rm{B}}\,{\rm{(}}{u}{n}{i}{t}{l}{e}{s}{s}{\rm{)}}{\rm{=}}\frac{\lceil {}^{{\rm{18}}}{\rm{F}}\rceil {\rm{-}}{\rm{F}}{\rm{M}}{\rm{I}}{\rm{S}}{\rm{O}}\,{\rm{u}}{\rm{p}}{\rm{t}}{\rm{a}}{\rm{k}}{\rm{e}}\,{\rm{(}}\frac{{\rm{k}}{\rm{B}}{\rm{q}}}{{\rm{m}}{\rm{l}}}{\rm{)}}}{\lceil {}^{{\rm{18}}}{\rm{F}}\rceil {\rm{-}}{\rm{F}}{\rm{M}}{\rm{I}}{\rm{S}}{\rm{O}}\,{\rm{m}}{\rm{e}}{\rm{a}}{\rm{s}}{\rm{u}}{\rm{r}}{\rm{e}}{\rm{d}}\,{\rm{i}}{\rm{n}}\,{\rm{t}}{\rm{h}}{\rm{e}}\,{\rm{b}}{\rm{l}}{\rm{o}}{\rm{o}}{\rm{d}}\,{\rm{(}}\frac{{\rm{k}}{\rm{B}}{\rm{q}}}{{\rm{m}}{\rm{l}}}{\rm{)}}}$$

## Magnetic Resonance Imaging

### Anatomical imaging

Investigations were performed with a Signa HDxt 1.5 T (GE Healthcare) in the radiology department of university hospital of Caen. After a scout view, an axial T2w FLAIR (fluid attenuated inversion recovery) sequence was performed to assess vasogenic edema.

### PWI

Dynamic T2*-weighted EPI images were acquired 30 s prior to and during the first pass of an intravenous bolus injection of 0.1 mmol/kg of Gd-DOTA (Dotarem®, Guerbet, France) and thereafter 60 s (24 slices, 40 repetitions, slice spacing: 5.5 mm, pixel resolution 2.19 × 2.19 mm, TR/TE = 2375/60 ms). Variations of the T2* signal in the tissue, which are proportional to the concentrations of the contrast agent, were calculated with in-house macros based on ImageJ software as: ∆R_2_ * (t) = −ln(S(t)/S_0_), where S_0_ = the signal intensity before contrast agent injection, and S(t) = the signal intensity over time. Then, CBV maps were generated by integrating the area under the γ-variate fitted curves to avoid any effect of recirculation of the contrast agent. Images were then normalized by dividing CBV maps by the mean value of the normal-appearing contralateral side (so as to compute relative CBV maps). Thereafter, a 3D T1-weighted sequence was performed for tumor contrast enhancement (124 slices, slice spacing 1.5 mm, pixel resolution 1.01 × 1.01 mm, TR/TE = 9.3/3.6 ms).

### Mono-voxel ^1^H-MRS

A standard PRESS (point resolved spectroscopy) sequence was used to measure regional metabolic differences in tumor and non-tumor tissues at several echo times [35 ms (all VOI), 144 ms (all VOI), 288 ms (partial VOI), 432 ms (partial VOI) and 576 ms (partial VOI)] with mono-voxel MRS. Only 144 ms has been retained to avoid the impact of macromolecular resonances. The VOI were first placed on the hyperperfused area identified on Gd-enhanced T1w images (tumor active areas), followed by the contralateral, mirror site (non-tumor tissue). For each VOI, 2048 points were sampled with a frequency of 2500 Hz to obtain the NMR spectra. After a frequency correction of all spectra, residual water components and potential lipids (5.4 ppm) were suppressed with a HLSVD (Hankel-Lenczos singular value decomposition) filter (frequency band of 4.2 ppm to 7.5 ppm). Frequency bands of interest for each metabolite around the principal resonance peaks were defined: for choline [Cho (3.35–3.10ppm)]; creatine [Cr (3.10–2.88 ppm)] and N-acetyl aspartate [NAA (2.24–1.87 ppm)]. We defined a frequency band [lactate and lipid (1.87–0.68 ppm)] that includes resonance of lactate (doublet at 1.3 ppm), major lipid resonances at 1.3ppm [CH_2_ group] and at 0.9 ppm [CH_3_ group]. The selection of these bandwidths is in accord with previously described *in vitro* and *in vivo* NMR studies of brain metabolites^25^. Areas of each frequency band of interest were then expressed as a percentage of the total area of the real value of spectra from 0 to 5.9 ppm. These proportions of area were taken as an index for the concentration of the metabolites. Lactate and lipid are pooled as it is difficult to distinguish the lactate doublets from lipids at 1.3 ppm. Accordingly we chose not to dissociate these compounds and to use a scale expressed as a percentage of the total area in order to obtain more precise indices of concentration, albeit with lower specificities. Pre-processing of the spectra was carried out on JMRUI 5.0 software.

### MRI, MRS and PET imaging co-registration

The PMOD 3.1® image fusion software was used to co-register the MRS volume of interest, [^18^F]-FMISO, p_t_O_2_ maps and rCBV to the reference sequence 3D T1w-Gd. The co-registration process was performed automatically, by a trilinear interpolation with rigid matching and normalized mutual information.

### Definition of tumor region of interest

For the GBM group, the severely hypoxic areas were determined from segmented [^18^F]-FMISO with values of T/B ratio >1.2^26^. Active tumor areas were segmented manually on 3D T1w-Gd images with no necrotic foci (which is formerly defined as an anoxic region) and used to assess vascular abnormalities. For the nGBM subjects, defined by the absence of both an increased [^18^F]-FMISO uptake and, vascular abnormalities, the region of interest (ROI) corresponding to the FLAIR hyperintensities was taken as the tumor. The edema is defined as a region with hyperintensities on T2w images, which encompasses the tumor core but also invaded tissue that cannot be considered anymore as a healthy tissue.

### Conversion of FMISO uptake into absolute values of the partial pressure of oxygen (p_t_O_2_) in brain tissue

The mathematical model^12^ initially proposed to convert [^18^F]-FMISO images into absolute p_t_O_2_ maps is:

2 $${{\rm{p}}}_{{\rm{t}}}{{\rm{O}}}_{2}({\rm{mmHg}})=\,\frac{{\rm{c}}\,({\rm{a}}-{\rm{u}})}{{\rm{b}}-{\rm{a}}+{\rm{u}}}$$

where **u** is the uptake as assessed by the T/B ratio and **a**, **b** and **c** are tissue-dependent, reaction-specific parameters. Based on the model described by Toma-Dasu and colleagues in neck and head tumors, which are also hypoxic^12^, a, b and c were 10.9, 10.7 and 2.5 respectively.

### The novel approach to convert [^18^F]-FMISO to ptO2 maps

After co-registration of all images, a processing step was performed to segment normal appearing gray matter (NAGM) and normal appearing white matter (NAWM) so as to generate masks (SPM8 software). The edematous zones were avoided. Three patients are exemplified in Fig. S1. The median T/B value of [^18^F]-FMISO uptake was measured in NAGM and NAWM region and p_t_O_2_ was fixed at 30 mmHg^27^ and 60 mmHg^28^ respectively. A least-square error minimization (Levenberg-Marquardt non-linear regression) was then performed under Matlab 2012b® to adjust **a**, **b** and **c** to the fixed p_t_O_2_ in NAGM and NAWM respectively to calibrate the equation. Thereafter, we calculated p_t_O_2_ in the whole brain by the model based on the estimates in normal appearing regions.

### Statistical analyses

Box plots, linear and logarithmic regressions, Tuckey’s HSD test and ROC curves analysis were performed using JMP Pro10 software (SAS). Further statistical analyses are detailed in the legends to figures. Data are presented either as mean ± sd or median and extremes of range.

### Data availability

The datasets generated and/or analyzed during the study are available from the corresponding author on reasonable request.

## Electronic supplementary material

Figure S1

## References

[1] Spence AM (2008). Regional hypoxia in glioblastoma multiforme quantified with [^18^F] fluoromisonidazole positron emission tomography before radiotherapy: correlation with time to progression and survival. Clin. Cancer Res.

[2] Evans M (2010). The relationship among hypoxia, proliferation, and outcome in patients with de novo glioblastoma. Transl. Oncol.

[3] Gray LH (1953). The concentration of oxygen dissolved in tissues at the time of irradiation as a factor in radiotherapy. Br. J. Radiol..

[4] Chinot OL (2014). Bevacizumab plus radiotherapy–temozolomide for newly diagnosed glioblastoma. N. Engl. J. Med.

[5] Ling CC (2000). Towards multidimensional radiotherapy (MD-CRT): biological imaging and biological conformality. Int. J. Radiat. Oncol. Biol. Phys..

[6] Piroth MD (2012). Integrated boost IMRT with FET-PET-adapted local dose escalation in glioblastomas. Results of a prospective phase II study..

[7] Corroyer-Dulmont A (2015). Imaging modalities to assess oxygen status in glioblastoma. Front. Med..

[8] Carreau A (2011). Why is the partial oxygen pressure of human tissues a crucial parameter? Small molecules and hypoxia. J. Cell. Mol. Med..

[9] Chang J (2009). A robotic system for ^18^F-FMISO PET-guided intratumoral pO2 measurements. Med. Phys..

[10] South CP (2009). Dose prescription complexity versus tumor control probability in biologically conformal radiotherapy. Med. Phys..

[11] Bowen SR (2011). Characterization of PET hypoxia tracer uptake and tissue oxygenation via electrochemical modeling. Nucl. Med. Biol..

[12] Toma-Dasu I (2012). Dose prescription and treatment planning based on FMISO-PET hypoxia. Acta Oncol..

[13] Fleming IN (2015). Imaging tumour hypoxia with positron emission tomography. Br. J. Cancer..

[14] Collingridge DR (1999). Polarographic measurements of oxygen tension in human glioma and surrounding peritumoural brain tissue. Radiother. Oncol. J. Eur. Soc. Ther. Radiol. Oncol..

[15] Lally BE (2006). The interactions of polarographic measurements of oxygen tension and histological grade in human glioma. Cancer J..

[16] Boxerman JL (2006). Relative cerebral blood volume maps corrected for contrast agent extravasation significantly correlate with glioma tumor grade, whereas uncorrected maps do not. AJNR..

[17] Cher LM (2006). Correlation of hypoxic cell fraction and angiogenesis with glucose metabolic rate in gliomas using 18F-fluoromisonidazole, ^18^F-FDG PET, and immunohistochemical studies. J. Nucl. Med..

[18] Gerstner, E. *et al*. ACRIN 6684: Assessment of tumor hypoxia in newly diagnosed GBM using ^18^F-FMISO PET and MRI. Clin Cancer Res, May 16 (2016). 10.1158/1078-0432.CCR-15-2529 PMC5065740 27185374

[19] Gross MW (1995). Calibration of misonidazole labeling by simultaneous measurement of oxygen tension and labeling density in multicellular spheroids. Int. J. Cancer..

[20] Hirata K (2012). ^18^F-Fluoromisonidazole positron emission tomography may differentiate glioblastoma multiforme from less malignant gliomas. Eur J Nucl Med Mol Imaging..

[21] Bekaert L (2017). [18F]-FMISO PET study of hypoxia in gliomas before surgery: correlation with molecular markers of hypoxia and angiogenesis. Eur J Nucl Med Mol Imaging..

[22] Cher LM (2006). Correlation of hypoxic cell fraction and angiogenesis with glucose metabolic rate in gliomas using ^18^F-Fluoromisonidazole, ^18^F-FDG PET, and immunohistochemical studies. J Nucl Med..

[23] Yamamoto, Y. *et al*. Hypoxia assessed by ^18^F-Fluoromisonidazole positron emission tomography in newly diagnosed gliomas. **33**, 621–625 (2012). 10.1097/MNM.0b013e3283529984 22422099

[24] Valable S (2011). Complementary information from magnetic resonance imaging and ^18^F-fluoromisonidazole positron emission tomography in the assessment of the response to an antiangiogenic treatment in a rat brain tumor model. Nucl. Med. Biol..

[25] Govindaraju V (2000). Proton NMR chemical shifts and coupling constants for brain metabolites. NMR Biomed..

[26] Szeto MD (2009). Quantitative metrics of net proliferation and invasion link biological aggressiveness assessed by MRI with hypoxia assessed by FMISO-PET in newly diagnosed glioblastomas. Cancer Res..

[27] Hou H (2012). Dynamic changes in oxygenation of intracranial tumor and contralateral brain during tumor growth and carbogen breathing: A multisite EPR oximetry with implantable resonators. J. Magn. Reson..

[28] Stokes BT (1982). Traumatically induced alterations in the oxygen fields in the canine spinal cord. Exp. Neurol..

