# Supplementary material for: FMISO-PET-derived brain oxygen tension maps: application to glioblastoma and less aggressive gliomas
Source: Sci Rep. 2017 Aug 31;7:10210. doi: 10.1038/s41598-017-08646-y (PMC5579277; doi:10.1038/s41598-017-08646-y)
Supplement: Supplementary file 1 — Figure S1 [file 41598_2017_8646_MOESM1_ESM.doc]

**FMISO-PET-derived brain oxygen tension maps: application to glioblastoma and less aggressive gliomas**

Ararat Chakhoyan^1^, Jean-Sebastien Guillamo^1,2^, Solène Collet^1^, François Kauffmann^3^, Nicolas Delcroix^4^, Emmanuèle Lechapt-Zalcman^1,5^, Jean-Marc Constans^1,6^, Edwige Petit^1^, Eric T. MacKenzie^1^, Louisa Barré^7^, Myriam Bernaudin^1^, Omar Touzani^1^ and Samuel Valable^1^.


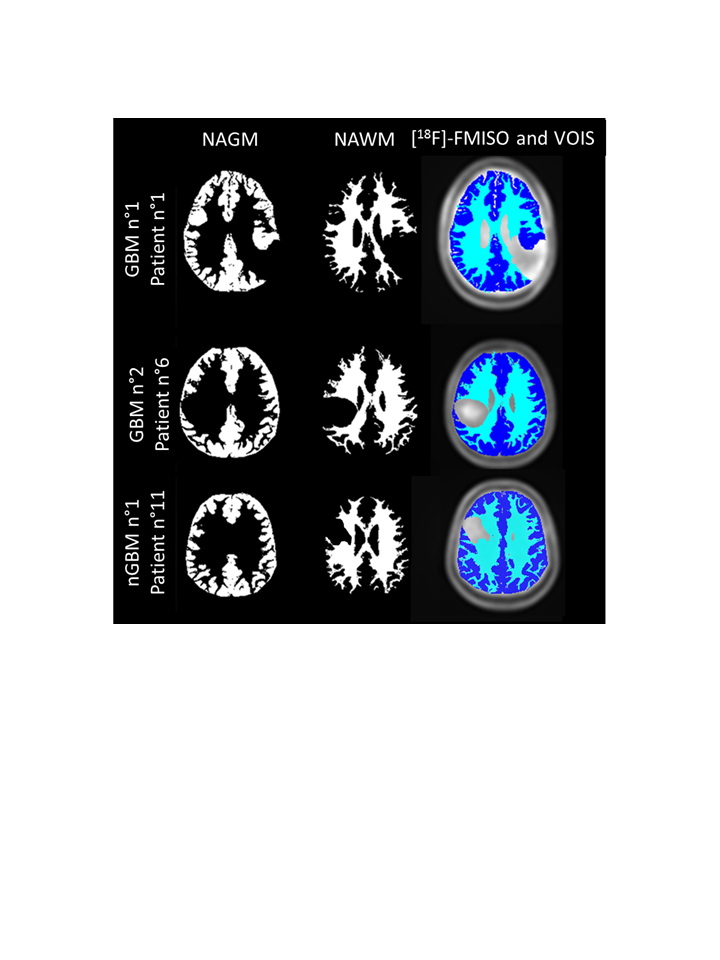


**Figure S1:** Representative images of the statistical segmentation (SPM8) of gray (GM) and white matter (WM) outwith the tumor region based on 3D-T1w post Gadolinium images. The edema region was used to mask the tumor area. Labeled GM and WM masks converted to overlay the [^18^F]-FMISO maps to calculate the uptake of radiotracer.
